# Transmembrane protein 100 is expressed in neurons and glia of dorsal root ganglia and is reduced after painful nerve injury

**DOI:** 10.1097/PR9.0000000000000703

**Published:** 2018-12-26

**Authors:** Hongwei Yu, Seung Min Shin, Fei Wang, Hao Xu, Hongfei Xiang, Yongsong Cai, Brandon Itson-Zoske, Quinn H. Hogan

**Affiliations:** a Department of Anesthesiology, Medical College of Wisconsin, Milwaukee, WI, USA; b Zablocki Veterans Affairs Medical Center, Milwaukee, WI, USA; c Medical Experiment Center, Shaanxi University of Chinese Medicine, Xianyang, Shaanxi, PR of China; d Department of Orthopedic Surgery, Affiliated Hospital of Qingdao University, Qingdao, PR of China; e Xi'an Jiaotong University Health Science Center, Xi'an, Shaanxi, PR of China

**Keywords:** Transmembrane protein 100, Dorsal root ganglia, Primary sensory neurons, Satellite glial cells, Microglia, Inflammatory pain, Neuropathic pain

## Abstract

**Introduction:**

Tmem100 modulates interactions between TRPA1 and TRPV1. The cell specificity of Tmem100 expression in dorsal root ganglia (DRGs) is not well defined, nor is the effect of peripheral nerve injury on Tmem100 expression.

**Objective:**

This study was designed to determine the cell specificity of Tmem100 expression in DRG and its subcellular localization, and to examine how Tmem100 expression may be altered in painful conditions.

**Methods:**

Dorsal root ganglion Tmem100 expression was determined by immunohistochemistry, immunoblot, and quantitative real-time PCR, and compared between various experimental rat pain models and controls.

**Results:**

Tmem100 is expressed in both neurons and perineuronal glial cells in the rat DRG. The plasma membrane and intracellular localization of Tmem100 are identified in 83% ± 6% of IB4-positive and 48% ± 6% of calcitonin gene-related peptide–positive neurons, as well as in medium- and large-sized neurons, with its immunopositivity colocalized to TRPV1 (94% ± 5%) and TRPA1 (96% ± 3%). Tmem100 is also detected in the perineuronal satellite glial cells and in some microglia. Tmem100 protein is significantly increased in the lumbar DRGs in the complete Freund adjuvant inflammatory pain. By contrast, peripheral nerve injury by spinal nerve ligation diminishes Tmem100 expression in the injured DRG, with immunoblot and immunohistochemistry experiments showing reduced Tmem100 protein levels in both neurons and satellite glial cells of DRGs proximal to injury, whereas Tmem100 is unchanged in adjacent DRGs. The spared nerve injury model also reduces Tmem100 protein in the injured DRGs.

**Conclusion:**

Our data demonstrate a pain pathology–dependent alteration of DRG Tmem100 protein expression, upregulated during CFA inflammatory pain but downregulated during neuropathic pain.

## Abstract 2

Supplemental Digital Content is Available in the Text.

## 1. Introduction

Peripheral nerve injury often causes chronic neuropathic pain, for which the specific underlying mechanisms typically are uncertain, and available therapies are currently inadequate.^6^ Pain perception results from the generation of action potential trains in the periphery, which are conveyed to the central nervous system (CNS) by primary sensory neurons, which have cell bodies in the dorsal root ganglion (DRG). Nerve injury–induced malfunction of the primary sensory neurons and surrounding glial cells is a well-established source of chronic neuropathic pain.^13^ Identification of the altered gene expression and associated molecular pathways involving primary sensory neurons and glial cells that lead to neuropathic pain will be required for successful generation of specific analgesic therapies.

Transmembrane protein 100 (Tmem100) is a recently identified pain-signaling modulator expressed by DRG nociceptive neurons, which is found in regulating pain sensation by modulating association of TRPA1 and TRPV1,^59^ both of which are important mediators of pain in sensory pathways.^6,13^ Tmem100 modulates TRPA1 activity by decreasing TRPA1–TRPV1 interaction. Tmem100 deletion selectively in sensory neurons leads to a reduced TRPA1 but not TRPV1-mediated mechanical hyperalgesia in mouse inflammation pain.^59^ Also, a short TRPA1-binding peptide derived from the Tmem100 protein C-terminus shows analgesic in models of inflammatory pain and chemotherapy-induced pain in mice.^59^ These results demonstrate the complex and divergent influences of Tmem100 and associated signal transduction pathways in modulating pain and reveal them as potential targets for pain therapy.^60^

Tmem100 is reported to be predominantly localized in calcitonin gene-related peptide (CGRP)-positive peptidergic neurons in mouse DRG and trigeminal ganglia, in which its expression is increased in inflammatory pain.^12,59^ However, the influence of painful peripheral nerve injury upon Tmem100 expression has not been determined. Gene expression during neuropathic and inflammatory pain can be distinct.^32,37,50,64^ For instance, IB4- and CGRP-positive small-sized DRG neurons are either functionally activated or increased in proportion in various inflammatory pain models.^1,9,18,30,66,71,72^ Complete Freund adjuvant (CFA) inflammation increases CGRP in the innervating DRG, and the proportion and size of DRG neurons with detectable CGRP is increased after inflammation ascribed to increased CGRP release,^54,63^ and more IB4 neurons have detectable CGRP expression after inflammation.^63^ By contrast, IB4- and CGRP-positive neuron populations are often decreased significantly because of peripheral nerve axotomy in neuropathic pain.^17,25,29,35,44^ Inflammation and nerve injury can induce different effects on the expression and functions of several ion channels in the primary sensory neurons, including sodium channels and voltage-gated calcium channels.^22,36,39,64^

This study was designed to address these gaps in understanding the role of Tmem100 in painful conditions, and also to consider the therapeutic potential of Tmem100 as a target for treating chronic neuropathic pain. Because the cell specificity of Tmem100 expression has not been fully examined, we determined Tmem100 expression in the different cell populations of adult rat DRGs. We further characterized Tmem100 expression in the CFA-inflammatory pain model and in peripheral nerve injury–induced neuropathic pain in rats. We found Tmem100 protein expression in the full range of primary sensory neuron populations in adult rat DRG. Tmem100 is also robustly detected in the perineuronal satellite glial cells (SGCs) and a few microglia. Pain induces alteration of Tmem100 expression in a pathology-dependent manner, upregulated after CFA-inflammatory pain but dramatically downregulated in the injured DRGs after peripheral nerve injury.

## 2. Methods

### 2.1. Animals

Adult male Sprague–Dawley rats weighing 100 to 125 g body weight (Charles River Laboratories, Wilmington, MA) were used. All animal experiments were performed with the approval of the Zablocki VA Medical Center Animal Studies Subcommittee and Medical College of Wisconsin Institutional Animal Care and Use Committee (Permit number: 3690-03) in accordance with the National Institutes of Health Guidelines for the Care and Use of Laboratory Animals. Animals were housed individually in a room maintained at constant temperature (22 ± 0.5°C) and relative humidity (60% ± 15%) with an alternating 12-hour light–dark cycle. Animals were given ad libitum access to water and food throughout the experiment, and all efforts were made to minimize suffering.

### 2.2. Generation of experimental rat pain models

Inflammatory pain was induced by subcutaneous intraplantar injection of a single dose of CFA, performed as described previously.^24^ In brief, on day 0, after baseline behavior testing (von Frey, Pin, and Heat), rats were anesthetized briefly with isoflurane (4% induction and 2% maintenance), and their right foot was swabbed with ethanol, followed by injection of 100 μL of CFA (Sigma-Aldrich, St. Louis, MO) at a concentration of 1.0 mg/mL or saline subcutaneously in the plantar surface of the right hind paw.^55^ Behavior was evaluated thereafter every 2 days until 10 days after CFA.

Two rat neuropathic pain models were used, the spinal nerve ligation (SNL) at the fifth lumbar (L5) level and the spared nerve injury (SNI) model. These were performed as previously described.^20,70^ Briefly, after baseline behavior testing (von Frey, Pin, and Heat), for unilateral right SNL, the right lumbar paravertebral region was exposed through a midline incision, and the L5 transverse process was removed to expose the L5 spinal nerve, which was then ligated with 6-0 silk sutures and severed approximately 5 mm distal to L5 DRG. To perform unilateral right SNI, an incision was made on the lateral midthigh, and the underlying muscles were separated to expose the right sciatic nerve, whereupon the tibial and common peroneal were individually ligated with 6-0 sutures and severed distally to the ligature, and 2–3 mm of each nerve was removed distal to the ligation. The sural nerve was preserved, and contact with it was avoided. After these procedures, muscle and skin were closed using 4.0 monofilament nylon sutures and wound clips. The rats were returned to their cages and monitored until they began ambulating. Behavior was evaluated thereafter weekly basis until 4 weeks after SNL or SNI.

Sham-operated rats were subjected to the procedures in the same manner but without nerve ligation or transection. Animals without surgery or manipulation are designated as naive rats.

### 2.3. Sensory behavioral testing

Animals were habituated in individual test compartments for at least 1 hour before each testing session. Behavioral tests by mechanical withdrawal threshold testing (von Frey test), noxious punctate mechanical stimulation (Pin test), heat nociception (Hargreaves test), and cold stimulation were performed as previously described.^19^ The von Frey test was performed using calibrated monofilaments (Patterson Medical, Bolingbrook, Illinois). Briefly, beginning with the 2.8-g filament, filaments were applied with just enough force to bend the fiber and held for 1 second. If a withdrawal response was observed, the next smaller filament was applied, and if no response was observed, the next larger was applied, until a reversal occurred, defined as a withdrawal after a previous lack of withdrawal, or vice versa. After a reversal event, 4 more stimulations were performed after the same pattern. The forces of the filaments before and after the reversal, and the 4 filaments applied after the reversal, were used to calculate the 50% withdrawal threshold.^11^ Rats not responding to any filament were assigned a score of 25 g. Mechanical hyperalgesia (Pin test) was initiated with noxious punctate mechanical stimulation, which was performed using the point of a 22-g spinal anesthesia needle that was applied to the center of plantar surface of the hindpaw with a force that indented the skin without penetration. Five applications were separated by at least 10 seconds, which was repeated after 2 minutes, making a total of 10 touches. For each application, the induced behavior was either a very brisk, simple withdrawal with immediate return of the foot to the cage floor, or a sustained elevation with grooming that included licking, chewing, and/or shaking, which lasted at least 1 second. We term this latter behavior a hyperalgesic response, which is specifically associated with place avoidance.^61^ A hyperalgesia score was quantified by tabulating hyperalgesia responses as a percentage of total touches. Heat nociception (Hargreaves test) was performed using a device designed for the purpose of identifying heat sensitivity (Paw Thermal Stimulator System; University Anesthesia Research & Development Group, San Diego, CA). Rats were placed on the temperature-regulated glass platform heated to 30°C, and the hind paw was stimulated with a radiant heat source (50-W halogen bulb) directed through an aperture. In SNI rats, the lateral plantar surface in the sural nerve territory was targeted. The time elapsed from initiation of the stimulus until withdrawal (withdrawal latency) was detected automatically by photocells. Each hind paw was tested 4 times, and the withdrawal latency values averaged. Cold sensitivity was determined using acetone, which was expelled from a syringe attached to PE220 tubing to make a meniscus that was touched to the plantar surface of the hind paw, such that the drop spread out on the plantar surface of the paw without contact of the tubing to the skin. Each hind paw was tested 3 times in alternating fashion. Any induced withdrawal was considered a positive response.^19^

### 2.4. Immunohistochemistry and analysis

For immunohistochemistry (IHC), animals were terminally anesthetized, the L5 DRGs as well as the corresponding levels of the spinal cords and the medial plantar skin of hindpaws were dissected, and tissues were postfixed in 10% buffered formalin, followed by paraffin embedding and sectioning. Immunohistochemistry double staining was performed to characterize cellular specificity and distribution of target molecules in sections, as previously described.^69^ In brief, 5-μm-thick sections were deparaffinized in xylene and rehydrated through graded alcohols, and treated by heat-induced epitope retrieval in 10-mM citrate buffer, pH 6.0∼7.0 (depending on the antibody used). Sections were first immunolabeled with the selected primary antibodies overnight at 4°C (Table 1). The specificity of the goat Tmem100 antibody (Santa Cruz Biotechnology, SCB) was tested by preincubating the antibody solution with the specific Tmem100 antigenic peptides (5 μg/mL; SCB) for 2 hours before immunostaining, as described previously.^58^ The specificities of the other antibodies, including a rabbit anti-Tmem100, a guinea pig anti-TRPV1, and a mouse anti-TRPA1, as well as antibodies of NKAα1, GFAP, and Iba1, used in this study, have been previously confirmed by immunostaining and immunoblotting.^2,56,58,59,62^ All antibodies were diluted in 1× phosphate-buffered saline, containing 0.1% Triton X-100 and 3% bovine serum albumin. Normal immunoglobulin G (from same species as the first antibody) replaced the first antibody for negative controls. The appropriate fluorophore-conjugated (Alexa 488 or Alexa 594, 1:2000) secondary antibodies (Jackson ImmunoResearch, West Grove, PA) were used to reveal the binding of immune complexes. The sections were washed 3 times (5 minutes each) with phosphate-buffered saline containing 0.05% Tween-20 between incubations. To stain nuclei, 1.0-μg/mL Hoechst 33342 (Life Technologies, Carlsbad, CA) was added to the secondary antibody mixture. The sections were examined, and images acquired on a Nikon TE2000-S fluorescence microscope (Nikon; El Segundo, CA), equipped with an Optronics QuantiFire digital camera and acquisition software (Ontario, NY), as well as filters suitable for selectively detecting the green, red, and blue fluorescence. For each comparative experiment, all images were acquired with identical settings for detector gain and exposure time under a 10× objective (0.5 numerical aperture at 2048 × 2048 pixel resolution) or 20× objective (0.3 numerical aperture at 1024 × 1024 pixel resolution). For double-label colocalization, images from the same section but showing different antigen signals were overlaid.

**Table 1:**
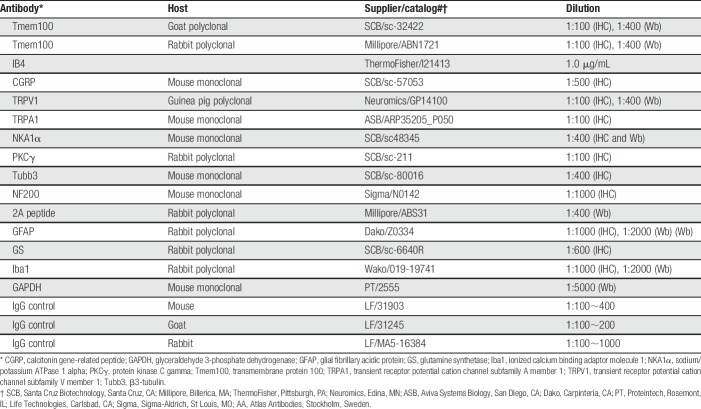
Primary antibodies and IgG controls used in this study.

Quantification of immunopositivity of Tmem100 and markers was performed as described previously.^35^ In brief, sections were immunostained for Tmem100 and counterstained with Tubb3 or Hoechst (n = 3 DRGs, L4/L5), as well as different markers as indicated. Immunohistochemistry pictures were selected from different levels at 10-section intervals. Cells were considered positive for a marker if their immunoreactive intensity was higher than mean plus 2-fold of SD of negative cells from the sections of negative controls. Percentages of Tmem100-positive cells were obtained by counting the number of Tmem100-positive cells and total positive cells of a given marker. All the counting and measurement, including the cross-sectional area and intensity (mean gray value) of Tmem100 and marker-positive neurons, was performed using Adobe Photoshop CC software. The background for each section was subtracted for intensity quantification. In addition, observer visual annotation (manual) for positive or negative staining was compared with the results of Photoshop software–assisted digital IHC quantification.

Intensity correlation analysis (ICA) was performed to determine colocalization of Tmem100 with neuronal plasma membrane (PM) marker sodium/potassium ATPase 1 alpha (NKA1α), SGC marker glial fibrillary acidic protein (GFAP) and glutamine synthetase (GS), and microglia marker–ionized calcium binding adaptor molecule 1 (Iba1) as previously described using an ImageJ 1.46r software plugin (http://imagej.nih.gov/ij).^33,58^ In brief, fluorescence intensity was quantified in matched region of interests (the green and red colors varied in close synchrony) for each pair of images. Mean background was determined from areas outside the section regions and was subtracted from each file. On the basis of the algorithm, in an image where the intensities vary together, the product of the differences from the mean (PDM) will be positive. If the pixel intensities vary asynchronously (the channels are segregated), then most of the PDM will be negative. The intensity correlation quotient (ICQ) is based on the nonparametric sign-test analysis of the PDM values and is equal to the ratio of the number of positive PDM values to the total number of pixel values. The ICQ values are distributed between −0.5 and +0.5 by subtracting 0.5 from this ratio. In random staining, the ICQ approximates 0. In segregated staining, ICQ is less than 0, whereas for dependent staining, ICQ is greater than 0.^58^

Tmem100 immunofluorescent intensity was quantified and compared between the ipsilateral and contralateral sides in the dorsal horn of lumbar spinal cord cross sections from SNL animals using ImageJ as described previously.^69^ In brief, the fluorescence intensity values of ipsilateral and contralateral sides were acquired along a line positioned between the dorsal root entry zone (DREZ), defining the entry surface of primary afferent into the spinal cord,^49^ and the central canal in the contralateral and ipsilateral sides of the section (line scan function, scan width 50 pixels). The intensity across the full length of this line was then determined for each section. The average intensity of Tmem100 staining in the superficial dorsal horn (SDH), defined as the region from DREZ to IB4-labeled laminae II inner edge (IIi), was compared between the ipsilateral and contralateral sides.

Immunocytochemistry (ICC) of Tmem100 expression was performed on rat L4 and L5 DRG cultures dissociated from naive rats as described previously.^70^ Dissociated DRG cells were plated on glass coverslips coated with poly-d-lysine (10 μg/mL; Sigma-Aldrich) for 15 hours and then fixed in 4% paraformaldehyde for 10 minutes. Fixed cells were processed for immunolabeling with a goat Tmem100 (1:100) and a rabbit GFAP (1:1000) or a mouse Tubb3 (1:2000) antibodies at 4°C for overnight, followed by the appropriate fluorophore-conjugated secondary antibodies. After immunostaining and washing, the coverslips were mounted onto glass slides for microscopic observation as above. A time-differential attachment protocol for astrocyte isolation was adapted to establish neuron-free SGC culture.^8^ Initially, the dissociated DRG cells were plated on culture dishes for 4 hours for SGC attachment, and then neurons that grow loosely attached to the top of mixed cultures were separated by hand-shaking of the culture flasks gently for 5 to 10 minutes, followed by replacing with new cultural medium.

### 2.5. Immunoblots

The lysates from DRG tissues and HEK293T cells transfected with pCMV-Tmem100 (rat ORF, RN210875; OriGene, Rockville, MD) or pCMV5.0, as well as various cell lines (all from ATCC, Manassas, VA) of F11 (rat DRG neurons), BV2 (mouse microglia), C6 (rat astrocytes), A549 (human non–small-cell lung cancer [NSCLC]), and N2A (mouse brain neurons) were extracted using 1× RIPA buffer (20 mm Tris-HCl pH 7.4, 150 mm NaCl, 1% Nonidet P-40, 1% sodium deoxycholate, 0.1% SDS, with 0.1% Triton X100 and protease inhibitor cocktail). As positive and negative controls for Tmem100 expression, 293T cells transfected with the plasmid pCMV-Tmem100 or pCMV5.0 were extracted at the same time. To examine the subcellular localization of Tmem100, DRG tissues were fractionated to obtain PM and cytosolic fractions using the ProteoExtract Subcellular Proteome Extraction Kit (Millipore, Billerica, MA), which contains extraction buffers with ultrapure chemicals to ensure high reproducibility, protease inhibitor cocktail to prevent protein degradation, and benzonase nuclease to remove contaminating nucleic acids, according to the manufacturer's instructions. Protein concentration was determined using the Pierce BCA kit (ThermoFisher, Pittsburgh, PA). Equivalent protein samples were size separated using 10% or 4% to 20% SDS-PAGE gels (Bio-Rad Laboratories, Des Plaines, IL), transferred to 0.22-μm nitrocellulose membranes, and blocked for 1 hour in 5% skim milk. The blots were subsequently incubated overnight at 4°C with a goat anti-Tmem100 (1:400) or rabbit anti-Tmem100 (1:400), a rabbit anti-Iba1 (1:2000), a rabbit anti-GFAP (1:2000), a mouse monoclonal anti-NKA1α (1:400), a mouse monoclonal anti-β3-tubulin (tubb3, 1:400), and a mouse monoclonal anti-GAPDH (1:5000). To verify the band specificity of Tmem100 detection using a goat Tmem100 antibody, the antibody solution was preincubated with the specific Tmem100 antigenic peptides (5 μg/mL; SCB) for 2 hours before immunoblotting. Immunoreactive proteins were detected by Pierce enhanced chemiluminescence (ThermoFisher) after incubation for 1 hour with HRP-conjugated second antibodies (1:2000; Bio-Rad). Densitometry of bands of interest was performed using ImageJ v.1.46. Ratios of the band density of the target proteins to the sum of housekeeping gene (Tubb3 or GAPDH) and NKA1α band density were calculated and the percentage changes of target proteins in the experimental samples compared with those from the control samples.^35,62^

### 2.6. Quantitative real-time PCR analysis

Total RNA was extracted from DRGs using RNAeasy kit (Qiagen, Carlsbad, CA) and then treated with DNase I (Life Technologies). Complementary DNA (cDNA) was synthesized from amounts of RNA that were standardized for each experiment using the Superscript III first strand synthesis kit with random hexamer primers (Life Technologies). Quantitative PCR was performed using IQ Syber Green supermix (Bio-Rad) on a Bio-Rad CFX96 Real-time PCR Machine and specific intron-spanning primers from rat to quantify the mRNA levels of Tmem100 and GAPDH. Tmem100 primers: forward 5′-CAGGTTCTCTTTTGTGGGTTCTTC-3′, reverse 5′-CAATACTCTGGCTGGTCCTTCTCT-3′; and GAPDH primers: forward 5′-AGACAGCCGCAT CTTCTTGT-3′, reverse 5′-TGATGGCAACAATGTCCACT-3′. The thermal cycling conditions were 1 cycle at 95°C for 1 minute, 40 cycles at 95°C for 15 seconds, and 1 cycle at 60°C for 15 seconds. For each sample, 2 inter-run determinations were performed, and 2 replicates in each run were averaged. GAPDH, which has been verified as the least affected reference gene at the mRNA level in the setting of nerve injury in our previous study,^4^ was used as the housekeeping gene for calculation of fold differences in expression of Tmem100 mRNA. The relative amount of mRNA transcript in the samples of the axotomized L5-DRGs from L5-SNL rats and the ipsilateral L5-DRGs from CFA rats to control DRGs (contralateral) was measured by 2^−ΔΔC^_t_ calculation, where ΔΔC_t_ = (C_t Tmem100_ − C_t GAPDH_)_ipsi.L5-DRG_ − (C_t Tmem100_ − C_t GAPDH_)_contra. DRG._

### 2.7. Lentivector constructs and proliferation assay

Lentiviral transfer plasmids pEF1α-Tmem100 and pEF1α-EGFP were used to express Tmem100 and EGFP, respectively. A viral 2A bicistronic lentiviral plasmid for coexpressing rat Tmem100 and EGFP under the EF1α promoter was constructed, as described previously.^68^ Specifically, rat Tmem100 cDNA coding sequence (GenBank accession number, NM_001017479) was inserted into plasmid pEF1α-EGFP immediate downstream of EGFP and a viral 2A autocleavage (or ribosome-skipping) sequence from thoseaasigna virus 2A was then cloned in frame between EGFP and Tmem100 to generate pEF1α-EGFP2ATmem100. Lentivectors expressing EGFP (LV-EGFP) or coexpressing Tmem100 and EGFP (LV-Tmem100) were packaged using pEF1α-EGFP and pEF1α-EGFP2ATmem100 with packaging plasmid pCMVΔR8.74 and envelop plasmid pVSV-g, and viral titration, as previously reported.^68^ The titers were in the range of 1 × 10^8^ to 1 × 10^9^ transduction unit/mL. Cultured C6 astrocytes, BV2 microglia, and freshly isolated primary SGCs from naive rats grown to 50% confluence were infected by LV-EGFP or LV-Tmem100 in the presence of 8 μg of polybrene (Sigma-Aldrich) per mL at an optimized multiplicity of infection = 5. After infection at 37°C for 12 hours, the medium was replaced. Transduction efficiency was estimated under a fluorescent microscope by calculating the percentage of green cells out of a total of 200 counted cells. Transgene expression was analyzed by Western blots (C6 and BV2 cells) as described above, and cell proliferation was measured using a Cell Counting Kit-8 (CCK-8; Sigma-Aldrich) assay, according to the manufacturer's protocol. Briefly, LV-Tmem100– or LV-EGFP–infected cells (3000) were seeded into each well of 96-well culture plate with DMEM containing 2% FBS and cultured in an incubator at 37°C. At the time for measurement, 10 μL of CCK-8 reagent was added into each well of the 96-well culture plate, and culture was continued under the same temperature for 4 hours. Finally, the 96-well plate plates were shaken for 10 min, and the data of OD values were measured at 450 nm in a plate reader.

### 2.8. Statistics

Statistical analysis was performed with GraphPad PRISM 6 (GraphPad Software, San Diego, CA). Behavioral changes over baseline and between groups for von Frey and heat measurements were made using repeated-measures 2-way analysis of variance (ANOVA) and post hoc analysis with Bonferroni test. Pin test, which results in discrete numerical data without normal distribution, was analyzed with conservative nonparametric tests including Friedman test for ANOVA and Dunn test for post hoc analysis. The differences of the targeted gene expression by immunoblots, qPCR, SDH Tmem100 immunofluorescent intensity analysis, and CCK8 assay were compared with 1-way or 2-way ANOVA, 2-tailed unpaired *t* test, or Mann–Whitney test where appropriate. Results are reported as mean and SEM. Significances of ICQs of Tmem100 immunocolocalization with NKA, GFAP, GS, and Iba1 were analyzed by means of the normal approximation of the nonparametric Wilcoxon rank test, as described previously.^33,62^ *P* < 0.05 were considered statistically significant.

## 3. Results

### 3.1. Identification of Tmem100 expression in neurons and glial cells in rat dorsal root ganglion

**Figure 1.**
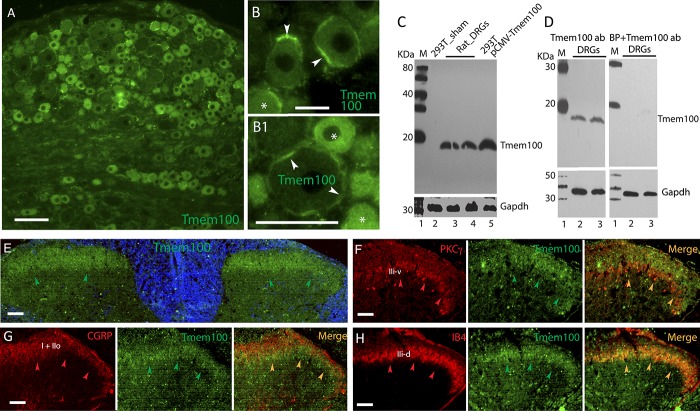
Tmem100 IHC profile and antibody preabsorption testing. Immunostained Tmem100 expression profile (A) with amplified images (B, B1) show the Tmem100 localization in intracellular (asterisks) and PM-like patterns (arrowheads) in DRG section from naive adult rat, using a goat anti-Tmem100 antibody, which detects a ∼17 KDa band on the lysates from DRGs (C, lanes 3 and 4) and pCMV-Tmem100 transfected 293T cells (C, lane 5) but not in the lysate from sham-transfected 293T cells (C, lane 2). Preincubation with antigenic peptide eliminates the band in immunoblot, suggesting specificity of detection (D). GAPDH immunoblotting (bottom panels in panels C and D) was used as the loading control. IHC using this antibody detected Tmem100 in SDH (E), Tmem100-IR signals symmetrical in the region from DREZ to PKCγ-labeled laminae II inner ventral edge (IIiv) (E and F) and concentrated on the CGRP-positive laminae I afferents (G) and IB4-positive laminae II fibers (H). Scale bar: 100 μm for all. CGRP, calcitonin gene-related peptide; DREZ, dorsal root entry zone; DRG, dorsal root ganglion; IHC, immunohistochemistry; PM, plasma membrane; SDH, superficial dorsal horn.

We first tested the suitability of 2 established antibodies for use in determining DRG expression of Tmem100 in rats. In DRGs from naive rats, IHC analysis on antigen-retrieved paraffin-sections detected a broad Tmem100 expression profile using a Tmem100 polyclonal antibody generated in goat against Tmem100 protein N-terminal 15aa fragment of mouse origin (Fig. 1A). This antibody has been verified for its specificity and applied to detect Tmem100 expression in mouse myenteric neurons and human hepatocellular carcinoma tissues in previous publications.^16,45^ Here, Tmem100 immunoreactivity (IR) stained the full profile of small- and middle-sized neurons, but in contrast displayed “ring-like” profiles in the case of large-sized neurons, in a pattern that could represent Tmem100 immunopositivity in neuronal membrane, SGCs, or both (Fig. 1A, B, B1). By immunoblot, the antibody revealed a clean band around ∼17 KDa (ie, slightly greater than apparent molecular mass of 14.5 KDa) as the target protein in the homogenates of DRGs from adult rats, which aligns with the size of the positive band in the lysates from Tmem100-cDNA–transfected HEK293T cells. This protein band was not detected in the sham-transfected cells (Fig. 1C). Preincubation with excess immunogenic peptide completely eliminated the band in immunoblots (Fig. 1D) and eliminated IHC staining in sections (data not shown). Tmem100-IR was also detected in the lumbar SDH (Fig. 1E), spanning from the DREZ to the laminae II inner ventral edge (IIiv) defined by the PKCγ-labeled intrinsic SDH neurons (Fig. 1F), therefore including the distribution of the terminations of the CGRP-positive primary afferents in laminae I (Fig. 1G) and the isolectin B4 (IB4)-positive afferents terminating in laminae II (Fig. 1H). Tmem100 showed no expression in intrinsic dorsal horn neurons labeled with PKCγ, suggesting that Tmem100 is synthesized in sensory neuron somata and transported to their central terminals in the DH superficial laminae. No immunostaining signals were observed when the first antibodies used were replaced by immunoglobulin G from the same species, or the second antibody was omitted, supporting the specific immunopositivity of IHC detection (Supplemental Fig. S1, available at http://links.lww.com/PR9/A37). Tmem100 was not identified in skin sections from the hind paw (data not shown) by our current IHC techniques. In a second antibody trial, we also used a rabbit Tmem100 polyclonal antibody raised by an immunogenic peptide corresponding to N-terminal 19aa sequence of mouse Tmem100 with the specificity verified in a previous report.^59^ This rabbit antibody showed sensitive Tmem100 protein detection as a clean ∼17 KDa band by immunoblot (Supplemental Fig. S2A, available at http://links.lww.com/PR9/A37). IHC with this antibody (Supplemental Fig. S2B, C, available at http://links.lww.com/PR9/A37) showed a pattern similar to that using the goat antibody, but seemed somewhat less sensitive for detecting Tmem100. Based on these results, our subsequent experiments used the goat polyclonal antibody to detect Tmem100 protein in the tissue sections and DRG-dissociated culture by IHC and ICC, and the rabbit polyclonal antibody was used for immunoblotting to detect Tmem100 expression in DRG tissue.

**Figure 2.**
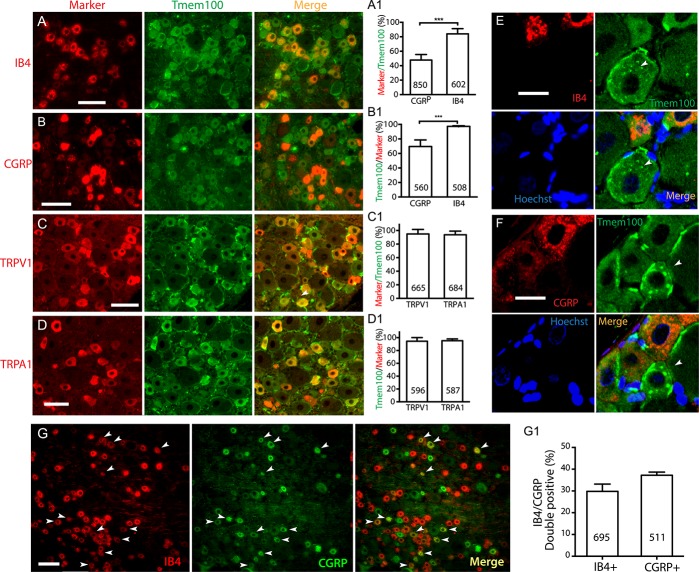
Double immunostaining of Tmem100 with a selection of nociceptive markers. Representative images show immunostaining of Tmem100 with IB4 (A), CGRP (B), TRPV1 (C), and TRPA1 (D) on DRG sections from naive rats. Bar charts of A1 to D1 in the right of panels of A to D show percentage of Tmem100 overlaid with each marker, respectively. The numbers in each bar is the numbers of total neurons analyzed from at least 4 animals per group. ***P < 0.001 for comparison between the percentage of Tmem100 overlaid with IB4 or CGRP. Some small neurons in sections show a characteristic punctate Tmem100 immunopositivity (E and F). Representative montage images (G) show about 30% to 40% (G1) double positivity of both IB4 (red) and CGRP (green) in DRG section from naive rats. The numbers in each bar is the numbers of neurons analyzed. Scale bars: 100 μm for all. CGRP, calcitonin gene-related peptide; DRG, dorsal root ganglion.

Tmem100 has been reported to be widely expressed in multiple tissues, but the cellular and subcellular distribution and specificity of Tmem100 expression in rat DRGs remain undefined. We therefore next determined the phenotype of small-sized neurons that express Tmem100 using IB4 as a marker for nonpeptidergic small neurons and CGRP as a marker for peptidergic small neurons. We observed high expression of Tmem100 in both IB4-positive and CGRP-positive neurons. An average of 97% ± 1% of Tmem100-positive neurons bound IB4 and 83% ± 6% of IB4-positive neurons expressed Tmem100. On average, 74% ± 13% of Tmem100-positive neurons expressed CGRP, whereas 48% ± 6% of CGRP-positive neurons expressed Tmem100 (Fig. 2A, A1, B, B1). In addition, Tmem100 is highly colocalized with TRPV1 (94% ± 5%) and TRPA1 (96% ± 3%) (Fig. 2C, C1, D, D1). Some small neurons showed a characteristic punctate Tmem100 immunopositivity suggesting subcellular localization (Fig. 2E, F). Approximately 30%-40% of IB4 or CGRP neurons are also positive for both CGRP and IB4 (Fig. 2G, G1) similar as previously reported,^48^ suggesting that Tmem100 can be expressed in IB4/CGRP double-positive neurons. These results together indicate that Tmem100 is abundantly expressed in the nonmyelinated C-fiber or lightly myelinated Aδ-fiber nociceptive neuron populations, with both neuronal membrane and intracellular localizations.

**Figure 3.**
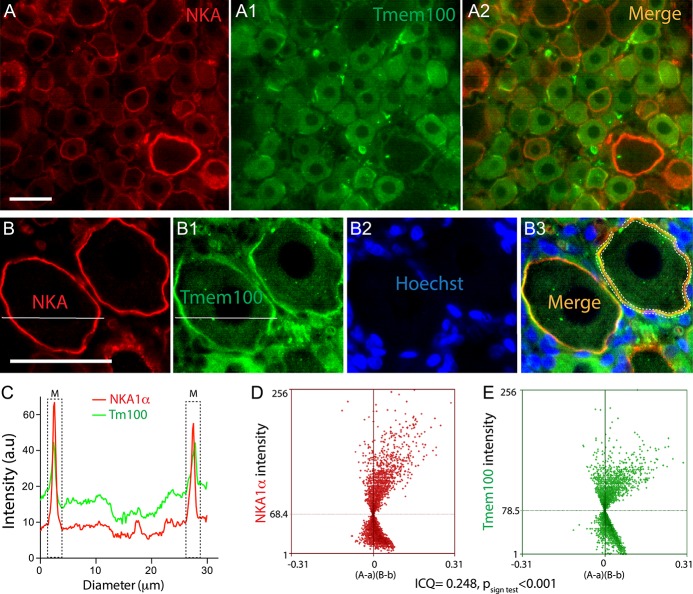
IHC delineation of Tmem100 localization to the PM in primary sensory neurons. Representative montage images show immunostaining of Tmem100 (green) with a canonical neuronal PM marker NKA1α (red) showing colabeling (yellow) in the merged image (A–A2, B–B3). Pixel intensity line-plot profiles of crossed sections clearly indicate neuronal PM localization of Tmem100 in the larger neurons (B, B1, C). ICA colocalization: Scatter plots for the region demarcated by the white dashed line in (B3) panel show strong right skewing for NKA1α (D) and Tmem100 (E). In (D, E), “A” is the intensity of Tmem100 while “a” is the average of these values, and “B” is the intensity of NKA1α while “b” is the average of these values. For this region, the intensity correlation quotient (ICQ) value is 0.248 (*P*_sign test_ < 0.001), indicating immunocolocalization. Scale bars: 100 μm for all. ICA, intensity correlation analysis; IHC, immunohistochemistry; PM, plasma membrane.

**Figure 4.**
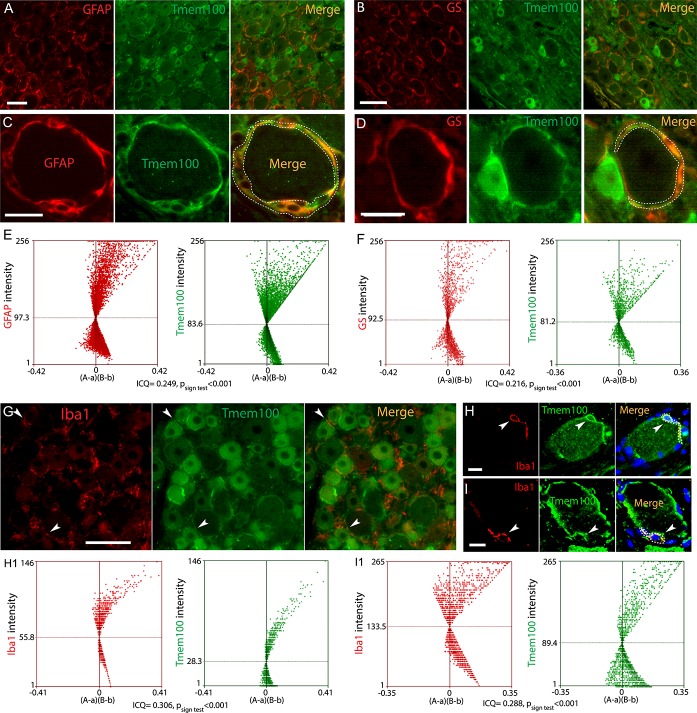
IHC analysis of Tmem100 immunocolocalization with glial cell markers. Representative montage images show immunocolocalization of Tmem100 with SGC marker GFAP (A and C) and GS (B and D). The ICA plots for the region demarcated by the white dashed line of Tmem100 and GFAP (E) or GS (F) were strongly skewed toward positive values with the calculated ICQ values were 0.249 (*P*_sign test_ < 0.001) for GFAP and 0.216 (*P*_sign test_ < 0.001) for GS both consistent with immunocolocalization. The scattered overlay was also found in the double labeling images of Tmem100 and Iba1 in naive DRG sections (G, H, I, arrowheads). The ICA plots for the region demarcated by the white dashed line of Tmem100 and Iba1 in ROIs in the panels (H and I) were strongly skewed toward positive values, consistent with immunocolocalization, 2 ROIs for Iba1 are 0.306 and 0.288 (*P*_sign test_ < 0.001 for both) (H1, I1). Scale bar: 100 μm for (A–G) and 20 μm for (H, I). DRG, dorsal root ganglion; GFAP, glial fibrillary acidic protein; GS, glutamine synthetase; ICA, intensity correlation analysis; ICQ, intensity correlation quotient; IHC, immunohistochemistry; ROIs, region of interests; SGC, satellite glial cell.

To identify whether the encircling profile of Tmem100 expression around large neurons consists of expression in neuronal PM or surrounding SGCs, we performed double immunolabeling of Tmem100 with either a canonical neuronal PM marker (NKA1α), SGC marker (GFAP or GS), or a microglia marker (Iba1), using antibodies for which the specificities were optimized (Supplemental Fig. 3, available at http://links.lww.com/PR9/A37), similar to our previous reports.^46,58,62,69^ Overlaid images of Tmem100 with NKA1α and pixel intensity line-plot profiles of cross-sections clearly identified neuronal PM localization of Tmem100 in these neurons (Fig. 3A–A2, B–B3, C). We also used the ICA method to analyze the extent of immunocolocalization between Tmem100 and NKA1α. Intensity correlation analysis plots of Tmem100 and NKA1α were strongly skewed toward positive values with the calculated ICQ value 0.248 (*P*_sign test_ < 0.001) (Fig. 3D), consistent with colocalization. These data thus suggest that at least a portion of the Tmem100 expression around the large-sized neurons is within the neuronal PM. In addition, however, partial or complete colocalization was also clearly delineated in the overlaid images of Tmem100 and GFAP or GS double immunolabeling, especially those encircling the large diameter neurons (Fig. 4A–D). To further verify the SGC expression of Tmem100, ICA was performed to analyze the immunocolocalization between Tmem100 and GFAP or GS. The ICA plots of Tmem100 and GFAP or GS were strongly skewed toward positive values (Fig. 4E, F), consistent with colocalization in SGCs, and the ICQ values were positive. Some overlay was also observed in double labeling images of Tmem100 with Iba1 in naive DRG sections (Fig. 4G–4I), with ICQ values indicating colocalization (Fig. 4H1, I1). These data thus suggest that Tmem100 is expressed in the perineuronal SGCs and in some of the DRG-resident microglia. Neuronal and SGC expression of Tmem100 was also confirmed by ICC double labeling of Tmem100 with markers on DRG-dissociated cultures (Supplemental Fig. S4, available at http://links.lww.com/PR9/A37).

Tmem100 expression has been identified in neural stem cells, neurons, astrocytes, and microglia of CNS origin.^7,10,27,28,38,52,57^ We also detected Tmem100 expression by immunoblot of lysates from various cell lines (Supplemental Fig. S5, available at http://links.lww.com/PR9/A37), including F11 (derived from rat DRG neurons), BV2 (from mouse microglia), C6 (from rat astrocytes), and N2A (from mouse brain cortex neurons). These observations indicate that a broad range of cell types express Tmem100. A weak Tmem100 band was also detected in the lysate of non-neuronal A549 cells (derived from human NSCLCs).

### 3.2. Increased dorsal root ganglion Tmem100 expression in inflammatory pain

Our experiments performed up to this point establish that Tmem100 is extensively expressed in neurons and glial cells in the normal rat DRG. We then asked how Tmem100 expression in the different cell populations is altered in painful conditions. In addition, because Tmem100 is differentially expressed in the PM of sensory neurons, we separately quantified Tmem100 in the NKA1α-enriched membrane fraction vs the NKA1α-deficient soluble fraction, hereafter referred to as PM and cytosolic fractions. These were extracted from the lumbar DRG tissues followed by immunoblotting of Tmem100 protein levels in these fractions.

**Figure 5.**
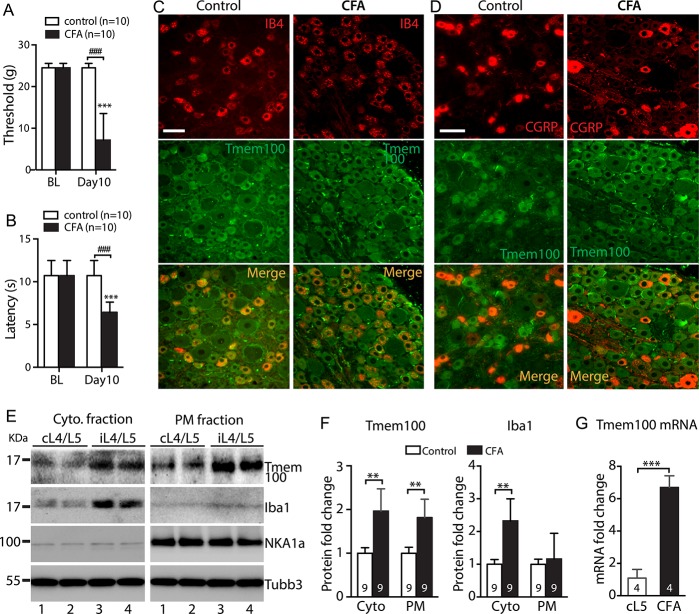
Behavior and Tmem100 expression in CFA inflammatory pain. Rats with CFA injection developed mechanical allodynia (von Frey, A) and Heat (B). ***P < 0.001for comparison to baseline (BL) and ###*P* < 0.001 for comparison between groups at the 10 days after injection. Representative montage IHC images shows the profiles of double immunolabeling of Tmem100 with IB4 (C) and CGRP (D) on the contralateral (contra.) L5 DRG of CFA and ipsilateral (ipsi.) L5 DRG at the 10 days after CFA. Scale bars: 100 μm for all. The NKA1α-eliminated cytosol and NKA1α-enriched PM protein fractions were extracted from the DRG tissues 10 days after CFA and control samples (E), and subjected to immunoblotting as shown in the representative immunoblots of Tmem100, Iba1, NKA1α, and Tubb3 of cytosolic fractions (left) and PM fractions (right), respectively. Bar charts are the results of densitometry analysis of immunoblots (F) and qPCR of Tmem100 transcript in DRG (G), **P < 0.01 and ***P < 0.001. The number in each bar is the number of analyzed DRGs per group. CFA, complete Freund adjuvant; GRP, calcitonin gene-related peptide; DRG, dorsal root ganglion; IHC, immunohistochemistry; PM, plasma membrane.

We used peripheral injection of CFA as a model of inflammatory pain. As expected, inflammatory pain was successfully induced by intraplantar injection of CFA in the right hindpaw, as demonstrated by marked circumferential edema and redness of injected hindpaw and the significantly reduced threshold for withdrawal from mild mechanical stimuli (von Frey) and PWL when compared with animals receiving only saline injection (Fig. 5A, B). Signs of inflammation lasted throughout the 10-day testing duration.

By semiquantitative IHC staining, a previous report observed an increase in the percentage of Tmem100-expressing DRG neurons in the CFA-inflammatory pain mouse model.^59^ We also observed an increased Tmem100 expression profile after CFA using IHC (Fig. 5C, D). Tmem100 is a putative PM-localized protein, but its subcellular localization by immunoblot has not been investigated. To gain information on the association and alteration of Tmem100 localization, the NKA1α-enriched membrane fraction and NKA1α-lacking soluble fraction were extracted from the lumbar DRG tissues harvested 10 days after CFA injection. Immunoblot results showed that Tmem100 was found in both PM and cytosolic fractions in the control DRGs, and that Tmem100 level from the DRGs ipsilateral to CFA injection was significantly higher in both fractions than those of control tissues (Fig. 5E, F). No significant differences were noted for GFAP protein levels by Western blot analysis between the ipsilateral DRGs harvested at the 10 days after CFA and controls (Data not shown). Tmem100 mRNA level was also significantly increased in the DRGs ipsilateral to CFA injection (Fig. 5G). These data are consistent with a previous report^59^ showing an upregulation of Tmem100 protein level in DRGs after CFA-inflammatory pain. And the upregulation of Tmem100 expression is likely due to increased de novo biosynthesis from neurons because Tmem100 transcript is also upregulated.

### 3.3. Decreased dorsal root ganglion Tmem100 expression in neuropathic pain

**Figure 6.**
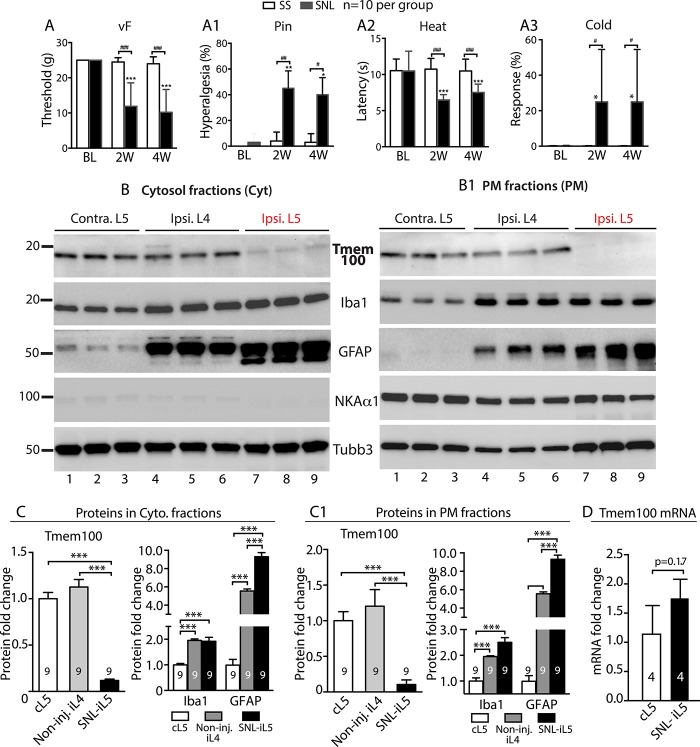
Behavior and immunoblot analysis of Tmem100 expression in DRGs after SNL. Rats with L5-SNL developed mechanical allodynia (von Frey, A), hyperalgesia (Pin, A1), Heat (A2), and Cold (A3) hypersensitivity. *P < 0.05, **P < 0.01, and ***P < 0.001 for comparison to baseline (BL) and #*P* < 0.05, ##*P* < 0.01, and ###*P* < 0.001 for comparison between groups at 2 and 4 weeks after SNL, respectively. The NKA1α-eliminated cytosol and NKA1α-enriched PM protein fractions were extracted from the DRG tissues at 28 days after L5-SNL and control samples, and subjected to immunoblotting as shown in the representative immunoblots of Tmem100, Iba1, GFAP, NKA1α, and Tubb3 of cytosol (B) and PM fractions (B1), respectively. Bar charts in the panels (C, C1) are the results of densitometry analysis of immunoblots (***P < 0.001). The number in each bar is the number of analyzed DRGs per group. Bar chart in the panel (D) is the result of qPCR quantification of Tmem100 mRNA in the L5 DRGs from ipsilateral (i) and contralateral (c) to SNL at 28 days after injury (n = 4 per groups). DRG, dorsal root ganglion; GFAP, glial fibrillary acidic protein; PM, plasma membrane; SNL, spinal nerve ligation.

After nerve injury by SNL, sensory testing revealed the expected pain behavior, which included reduced threshold for withdrawal from von Frey testing representing mechanical allodynia, exaggerated, and sustained responses to noxious mechanical stimulation (Pin testing) representing mechanical hyperalgesia, and increased sensitivity to heat and cold stimulation representing thermal hypersensitivity. These changes lasted throughout the 4-week testing interval (Fig. 6A–A3).

Immunoblot analysis of Tmem100 levels using DRGs harvested 28 days after SNL revealed a clean band at ∼17 KDa in the PM and cytosolic fractions extracted from the contralateral DRGs. However, the Tmem100 protein level in the ipsilateral L5 DRGs were significantly decreased to 11.3% ± 1.2% in the PM fraction and 10.5% ± 6.4% in the cytosolic fractions, normalized to the corresponding fractions from contralateral L5 DRGs, whereas Tmem100 levels in both fractions from the noninjured L4 DRGs did not change compared with the contralateral levels (n = 9 DRGs in each group). Iba1 (a PM and cytoplasm colocalized protein) and GFAP (a cytoskeleton protein) were found in both PM and cytosolic fractions, and their levels were increased not only in the injured L5 DRGs but also in the noninjured L4 DRGs, compared with the contralateral DRGs (Fig. 6B, B1, C, C1). These results indicate that the spared L4 DRGs, although not axotomized, developed changes resembling inflammatory responses, which has been reported previously.^34,44^ Elevated Iba1 and GFAP protein levels reflect the proliferation SGCs and resident microglia, so the lack of Tmem100 elevation suggests that Tmem100 expression in these glial cells of axotomized L5-DRGs was also likely suppressed. We also examined Tmem100 mRNA expression in the axotomized L5-DRGs at 4 weeks after SNL compared with the contralateral DRGs by qPCR. In contrast to protein levels, we observed slightly elevated but not significant changes of Tmem100 mRNA levels in the injured L5 DRGs (Fig. 6D).

**Figure 7.**
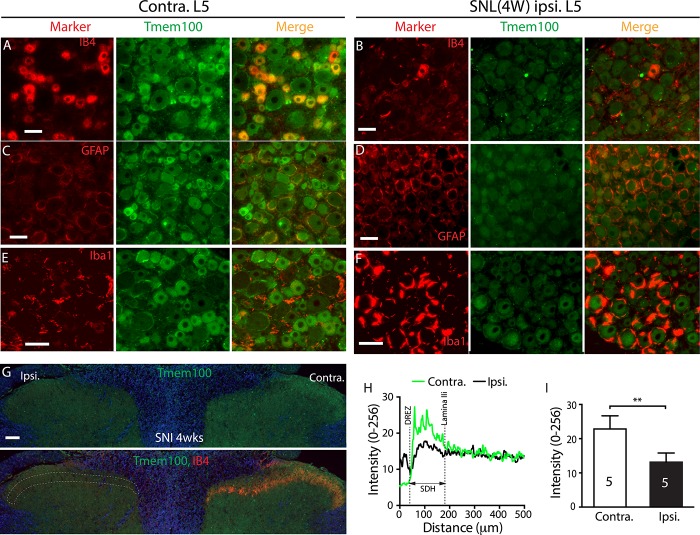
IHC analysis of Tmem100 expression following SNL nerve injury. Representative montage IHC images shows normal profiles of double immunolabeling of Tmem100 with IB4 (A), GFAP (C), and Iba1 (E) on the contralateral (contra.) L5 DRG of SNL rat, while the ipsilateral (ipsi.) L5 DRG at the 4 weeks after SNL show apparently decreased Tmem100 profile in the neuronal and perineuronal glial cells with diminished IB4 binding (B) and increased GFAP (D) and Iba1 (F) staining. The panel (G) shows Tmem100-IR is also decreased in the ipsi. SDH compared with the contra. side; parallel with decreased ipsi. IB4 staining. Scale bars: 100 μm for all. Quantification of Tmem100 immunofluorescence was performed along lines on the contra. and ipsi. sides from the DREZ to central canal, producing traces of intensity (H). The average fluorescence intensity of Tmem100 staining in the SDH, defined a region from DREZ to IB4-labeled laminae II inner edge (IIi) is decreased (**P < 0.01), compared to the contra. side (I). The number in each bar is the number of analyzed animals (3 sections per rat). DREZ, dorsal root entry zone; DRG, dorsal root ganglion; GFAP, glial fibrillary acidic protein; IHC, immunohistochemistry; SDH, superficial dorsal horn; SNL, spinal nerve ligation.

The concordant neuron and SGC downregulation of Tmem100 expression in the axotomized L5-DRGs was verified by IHC analysis performed on DRGs 4 weeks after SNL. These IHC images showed that Tmem100-IR intensity for glial cells, and all neuronal size groups were diminished in the ipsilateral L5 DRGs (Fig. 7, cf. A, C, E in control to B, D, F in SNL). Injury-induced loss of Tmem100 in the DRG paralleled with loss of IB4 binding (and CGRP staining, data not shown) and increased GFAP and Iba1 immunostaining. As with our Western data, although GFAP and Iba1 protein staining was markedly increased, likely reflecting injury-induced neural inflammatory response, the Tmem100 protein was lost, further supporting diminished Tmem100 protein expression in both neurons and glial cells after axotomy. In parallel, Tmem100-IR in the ipsilateral lumbar SDH in SNL rats was significantly reduced (Fig. 7G–I), compared to that in the contralateral site.

**Figure 8.**
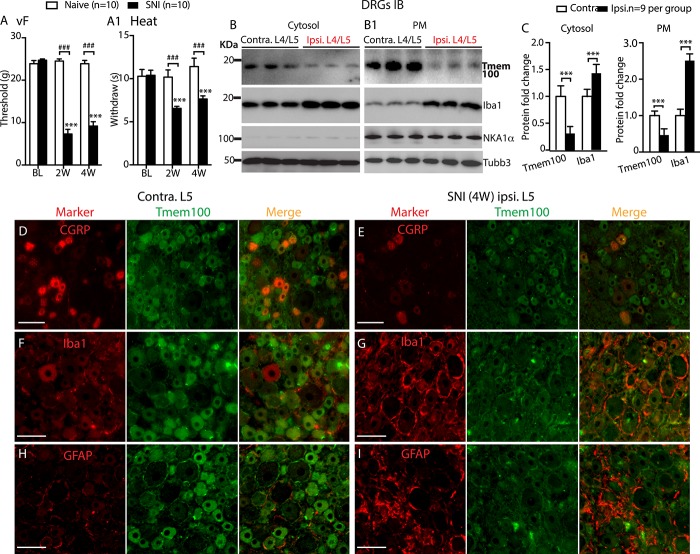
Behavior and immunoblot analysis of Tmem100 expression in DRGs following SNI. Rats with SNI developed mechanical allodynia (von Frey, A) and heat hypersensitivity (A1); ***P < 0.001 for comparison to baseline (BL) and ###*P* < 0.001 for comparison between groups at 2 and 4 weeks after SNI, respectively. The NKA1α-eliminated cytosol and NKA1α-enriched PM protein fractions were extracted from the contra. and ipsi. L4/L5 DRG tissues at 28 days after SNI, and subjected to immunoblotting. Representative immunoblots of Tmem100, Iba1, NKA1α, and Tubb3 in cytosol (B) and PM fractions (B1) are shown, respectively. Bar charts in the panel (C) are the results of densitometry analysis of immunoblots, as indicated. ***P < 0.0001 for comparison between groups. Representative montage IHC images shows normal profiles of double immunolabeling of Tmem100 with CGRP (D), Iba1 (F), and GFAP (H) on the contralateral (contra.) L5 DRG of SNI rat, while the ipsilateral (ipsi.) L5 DRG at the 4-week after SNI shows apparently decreased Tmem100 profile in the neuronal and perineuronal glial cells with decreased CGRP (E) and increased Iba1 (G) and GFAP (I) staining. Scale bars: 100 μm for all. CGRP, calcitonin gene-related peptide; DRG, dorsal root ganglion; GFAP, glial fibrillary acidic protein; IHC, immunohistochemistry; PM, plasma membrane; SNI, spared nerve injury.

To further confirm that peripheral nerve injury downregulates Tmem100 protein levels, we performed immunoblots from DRGs after injury by the SNI model of neuropathic pain, which results in DRGs containing commingled axotomized and noninjured neurons. These rats developed painful mechanical allodynia (von Frey) and hyperalgesia (Pin) (Fig. 8A, A1) during 4 weeks after SNI. Dorsal root ganglia processed for PM and cytosolic fractionation produced immunoblots that showed depressed Tmem100 protein levels in both PM and cytosolic fractions extracted from the SNI-DRGs compared with the control DRGs (contralateral) (Fig. 8B, C). Immunohistochemistry staining delineated downregulation profile of Tmem100 in both neurons and SGCs with apparently decreased CGRP staining and increased Iba1 and GFAP staining in DRG sections from SNI rats, compared with controls (Fig. 8D–I).

Together, these data indicate that painful peripheral nerve injury induces significant downregulation of Tmem100 protein expression in DRG neurons and glial cells, although the latter cell populations are highly proliferated and activated after nerve injury.

### 3.4. Overexpression of Tmem100 inhibits astrocyte, microglia, and satellite glial cell proliferation

**Figure 9.**
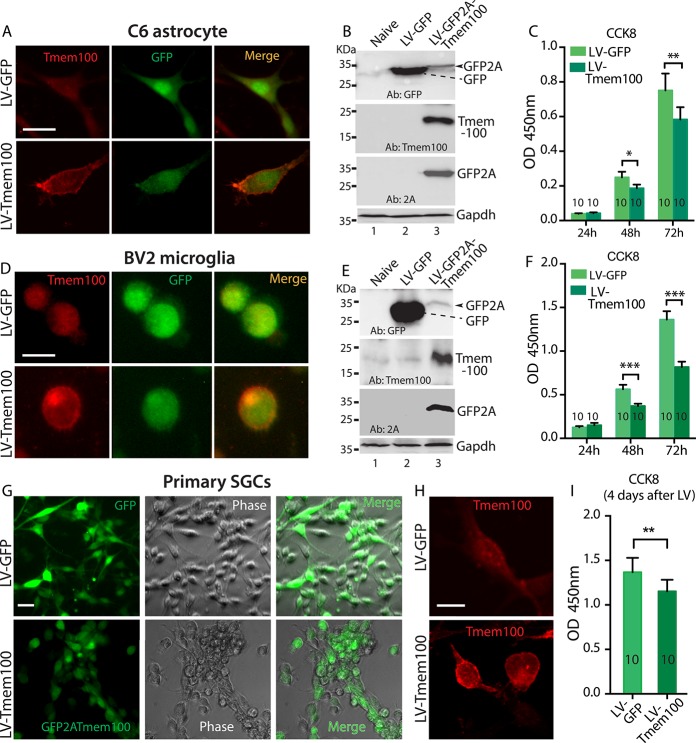
Effect of overexpression of Tmem100 on astrocyte, microglia, and SGC proliferation. EGFP or EGFP2ATmem100 (green) expression with ICC detected Tmem100 (red) and merged images as indicated in C6 astrocytes 4 days after transduction by LV-EGFP, MOI = 5 (top panels) and LV-Tmem100, MOI = 5 (bottom panels) (A, scale bar: 25 μm for all). Western blots show expression of EGFP (top), 2A (middle), and Tmem100 in C6 astrocytes transduced by LV-Tmem100 (B). Overexpression of Tmem100 significantly inhibits cell proliferation of C6 astrocytes, compared with the cells transduced by LV-EGFP control vector (C). EGFP or GFP2ATmem100 (green) expression with ICC detected Tmem100 (red) and merged images in BV2 microglia 4 days after transduction by LV-EGFP, MOI = 5 (top panels) and LV-Tmem100, MOI = 5 (bottom panels) (D, scale bar: 25 μm for all). Western blots show expression of EGFP (top), 2A (middle), and Tmem100 in BV2 microglia transduced by LV-Tmem100 (E). Overexpression of Tmem100 significantly inhibits cell proliferation of BV2 microglia, compared with the cells transduced by LV-EGFP control vector (F). Montage images (G) show EGFP or EGFP2ATmem100 (green), SGC morphology (phase), and merged images, as well as Tmem100 ICC (H) in the primary SGCs after transduction by LV-EGFP, MOI = 5 (top panels) and LV-Tmem100, MOI = 5 (bottom panels). Cell proliferation analyzed by CCK8 assay is significantly slower in the LV-Tmem100–transduced SGCs, compared with LV-EGFP–infected cells (I). *P < 0.05, **P < 0.01, and ***P < 0.001. The numbers in each bar in panels (C, F, I) indicate the repeats of CCK8 assay. ICC, immunocytochemistry; MOI, multiplicity of infection; SGC, satellite glial cell.

Previous works indicate a role of Tmem100 in controlling cell proliferation.^21,26,45,65^ Our data suggest that increased proliferation of DRG SGCs and resident microglia after nerve injury was associated with downregulation of SGC-Tmem100 in SNL and SNI rats. To test the hypothesis that Tmem100 might play a transregulator role for glial cell proliferation, we generated LV expressing Tmem100 or EGFP as control and infected C6 astrocytes and BV2 microglial cell lines (representative of DRG SGCs and microglia, respectively), which resulted in a successful transduction rates of >95% in both C6 and BV2 cell lines. CCK8 assay was used to detect the effect of proliferation after overexpression of Tmem100 or EGFP in these cell lines. Results showed that LV-mediated Tmem100 overexpression significantly inhibited C6 astrocytes (Fig. 9A–C) and BV2 microglia (Fig. 9D–F) proliferation, compared with LV-EGFP infected cells. Inhibition of cell proliferation by Tmem100 was further tested in the isolated neuron-free SGCs from naive rats. An approximately 90% transduction rate of SGCs was found by both LV-Tmem100 and LV-EGFP 4 days after addition of the vector. The growth rate of SGCs 4 days after infected by LV-Tmem100 was significantly slower than that of the SGCs infected by LV-EGFP (Fig. 9G–I).

## 4. Discussion

Tmem100 has been reported to be widely expressed in multiple mammalian tissues, with high abundance in lung and arterial endothelium.^26,41^ Tmem100 is also found in neurons and glial cells of the CNS and in the peripheral nervous system.^16,59^ Here, we report expression of Tmem100 in multiple cell types in the adult rat DRG. Specifically, we demonstrate that Tmem100 is expressed in the full range of primary sensory neuron populations, including the IB4-positive nonpeptidergic and CGRP-positive peptidergic small neurons, as well as the medium- and large-diameter neurons. Tmem100 is highly colocalized with TRPV1 and TRPA1 and is found in both PM and intracellular compartments in smaller neurons. In large neurons, Tmem100 is enriched in the PM, with immunopositivity in close apposition to surrounding SGCs that themselves robustly express Tmem100. This localization of Tmem100 expression in both the PM and cytosolic fractions is also verified by immunoblot analysis. Finally, we also identify Tmem100 expression in a small subset of resident microglia in the control DRG. Therefore, building on previous findings that characterize Tmem100 expression using EGFP as a marker based on a BAC Tmem100-EGFP mouse line,^59^ our results add new insights to the Tmem100 expression profile by directly detecting Tmem100 expression using a highly specific Tmem100 antibody. Although we detect Tmem100 in most IB4-neurons and in CGRP neurons in rat DRG using 2 distinct antibodies, a prior report^59^ described minimal expression of Tmem100 in IB4-binding neurons. It is reported that a substantial of nociceptive neurons in adult rat DRG is double positive for both IB4 and CRRP, but the overlay is much less in mouse.^48^ Thus, the discrepancy between 2 studies may be the result of different experimental conditions and approaches including antibody difference, IHC methods (cryosection vs paraffin-section with antigen retrieval process), and possible species differences between rat and mouse.^5,40,48^

Our data indicate that painful conditions are accompanied by divergent alterations of Tmem100 expression in a pathology-dependent manner, with upregulation after CFA-inflammatory pain, as in a previous study,^59^ but dramatic downregulation in the injured DRGs after peripheral nerve injury. Thus, the role played by Tmem100 in the development of pain likely differs between neuropathic and inflammatory mechanisms. This is reminiscent of a similar divergence of the effects of inflammation vs axotomy on other sensory neuron properties, including expression levels of voltage-gated Ca^2+^ and Na^+^ currents.^22,36,39,64^ The percentage of CGRP-positive DRG neurons is consistently increased in various inflammatory pain models, accompanied by functional activation of CGRP^+^ and IB4^+^ neurons.^1,9,18,30,66,71,72^ Our data cannot indicate if there is a causal link between increased number and/or functionally activated populations of CGRP^+^ and IB4^+^ neurons and the upregulated Tmem100 expression in inflammatory pain. In contrast to inflammatory pain, our data found a significant downregulation of Tmem100 protein in axotomized sensory neurons in the neuropathic pain models. The factors that trigger DRG neuronal Tmem100 depletion after nerve injury are unclear. One of the possibilities may be death of the axotomized neurons because IB4 binding (and CGRP staining) is also diminished.^14,17,23,35^ Further study is needed to determine how this change correlates temporally with the development of pain behavior after injury, and whether this nerve injury–induced Tmem100 downregulation would reverse in a longer timeframe because pain behavior and altered gene expression may eventually recover after nerve injury.^3,25^ In addition, the unaffected level of Tmem100 mRNA level in the axotomized DRGs suggests that the diminished Tmem100 protein expression after nerve injury may be the result of a translational repression of Tmem100 mRNA or rapid Tmem100 protein turnover, rather than DRG cell death after injury. It is reported that neuronal excitation in an in vitro coculture system suppresses astrocyte gene expression, including Tmem100,^27^ so neuronal hyperexcitability after nerve injury^51^ could be a contributing factor in Tmem100 loss after nerve injury.

Previous research has characterized the molecular function of Tmem100 in modulating TRPV1 and TRPA1 association in primary sensory neurons.^59^ However, there is as yet no evidence of TRPV1–TRPA1 interaction in SGCs. Thus, the presence of Tmem100 in SGCs adds another site for the potential roles of Tmem100 in neuropathic pain. Furthermore, a recent report found that Tmem100 is not restricted to the neurons coexpressing TRPV1 and TRPA1,^43^ suggesting that Tmem100 may serve other functions unrelated to these receptors, perhaps by interacting with additional nociceptive transducers.^60^

Although nerve injury results in proliferation and activation of glial cell populations, our data indicate that the normal expression of Tmem100 by these non-neuronal cells is nonetheless suppressed in this setting. There is growing evidence of the involvement of Tmem100 in controlling developmental proliferation and differentiation.^65^ For instance, Tmem100 is involved in the pathways activated by the TGFβ/BMPs/activin family of signaling molecules that are critical in developmental cellular differentiation.^15,31,42,47,53^ Tmem100 has also been linked to cell proliferation and apoptosis of various tumor cells and tumor-derived neuronal cells. For instance, Tmem100 expression is significantly downregulated in human hepatocellular carcinoma,^45^ NSCLC,^26^ and glioblastoma,^67^ whereas overexpression of Tmem100 in NSCLC cell lines inhibits tumor cell proliferation.^21,26^ Together, these studies suggest that Tmem100 may act as an important cell proliferation regulator. We also found that viral-mediated overexpression of Tmem100 in astrocyte and microglia cell lines and in SGCs isolated from rat DRG significantly blunt their proliferation in culture. These data suggest that Tmem100 likely plays a role in control SGC proliferation and also suggest that nerve injury–induced downregulation of SGC-Tmem100 by an undefined mechanism may possibly be correlated with increased SGC proliferation in animals with painful nerve injury. Although the direct effect of Tmem100 on SGC function is yet to be determined in vivo, downregulation of Tmem100 may be considered as a cause of SGC proliferation and pain after nerve injury. If this causal link is confirmed, then overexpression Tmem100 in SGCs might provide analgesia after nerve injury by reducing SGC proliferation/activation. By contrast, the opposite may be true in inflammatory pain, for which downregulating its expression or interfering in its function in neurons may be analgesic.

In conclusion, this study reports that Tmem100 is expressed in multiple cell types in rat DRGs, including the full range of sensory neuron populations, as well as SGCs and resident microglia. Because Tmem100 protein expression is diminished in neuropathic pain models, manipulation of this signaling pathway may be a means of controlling clinical neuropathic pain.

## Disclosures

The authors have no conflict of interest to declare.

## Appendix A. Supplemental digital content

Supplemental digital content associated with this article can be found online at http://links.lww.com/PR9/A37.

## References

[1] Aoki Y Ohtori S Takahashi K Ino H Takahashi Y Chiba T Moriya H Innervation of the lumbar intervertebral disc by nerve growth factor-dependent neurons related to inflammatory pain. Spine (Phila Pa 1976) 2004;29:1077–81. 15131432 10.1097/00007632-200405150-00005

[2] Avenali L Narayanan P Rouwette T Cervellini I Sereda M Gomez-Varela D Schmidt M Annexin A2 regulates TRPA1-dependent nociception. J Neurosci 2014;34:14506–16. 25355205 10.1523/JNEUROSCI.1801-14.2014 PMC4212057

[3] Bailey AL Ribeiro-da-Silva A Transient loss of terminals from non-peptidergic nociceptive fibers in the substantia gelatinosa of spinal cord following chronic constriction injury of the sciatic nerve. Neuroscience 2006;138:675–90. 16413131 10.1016/j.neuroscience.2005.11.051

[4] Bangaru ML Park F Hudmon A McCallum JB Hogan QH Quantification of gene expression after painful nerve injury: validation of optimal reference genes. J Mol Neurosci 2012;46:497–504. 21863315 10.1007/s12031-011-9628-x PMC3273664

[5] Barabas ME Kossyreva EA Stucky CL TRPA1 is functionally expressed primarily by IB4-binding, non-peptidergic mouse and rat sensory neurons. PLoS One 2012;7:e47988. 23133534 10.1371/journal.pone.0047988 PMC3485059

[6] Basbaum AI Bautista DM Scherrer G Julius D Cellular and molecular mechanisms of pain. Cell 2009;139:267–84. 19837031 10.1016/j.cell.2009.09.028 PMC2852643

[7] Berezovsky AD Poisson LM Cherba D Webb CP Transou AD Lemke NW Hong X Hasselbach LA Irtenkauf SM Mikkelsen T deCarvalho AC Sox2 promotes malignancy in glioblastoma by regulating plasticity and astrocytic differentiation. Neoplasia 2014;16:193–206, 206.e119–25. 24726753 10.1016/j.neo.2014.03.006 PMC4094829

[8] Bhat NR Shanker G Pieringer RA Investigations on myelination in vitro: regulation of 2,3'-cyclic nucleotide 3'-phosphohydrolase by thyroid hormone in cultures of dissociated brain cells from embryonic mice. J Neurochem 1981;37:695–701. 6268753 10.1111/j.1471-4159.1982.tb12543.x

[9] Breese NM George AC Pauers LE Stucky CL Peripheral inflammation selectively increases TRPV1 function in IB4-positive sensory neurons from adult mouse. PAIN 2005;115:37–49. 15836968 10.1016/j.pain.2005.02.010

[10] Butovsky O Jedrychowski MP Moore CS Cialic R Lanser AJ Gabriely G Koeglsperger T Dake B Wu PM Doykan CE Fanek Z Liu L Chen Z Rothstein JD Ransohoff RM Gygi SP Antel JP Weiner HL Identification of a unique TGF-beta-dependent molecular and functional signature in microglia. Nat Neurosci 2014;17:131–43. 24316888 10.1038/nn.3599 PMC4066672

[11] Chaplan SR Bach FW Pogrel JW Chung JM Yaksh TL Quantitative assessment of tactile allodynia in the rat paw. J Neurosci Methods 1994;53:55–63. 7990513 10.1016/0165-0270(94)90144-9

[12] Chung MK Park J Asgar J Ro JY Transcriptome analysis of trigeminal ganglia following masseter muscle inflammation in rats. Mol Pain 2016;12. 10.1177/1744806916668526 PMC5066585 27702909

[13] Costigan M Scholz J Woolf CJ Neuropathic pain: a maladaptive response of the nervous system to damage. Annu Rev Neurosci 2009;32:1–32. 19400724 10.1146/annurev.neuro.051508.135531 PMC2768555

[14] Duraku LS Hossaini M Hoendervangers S Falke LL Kambiz S Mudera VC Holstege JC Walbeehm ET Ruigrok TJ Spatiotemporal dynamics of re-innervation and hyperinnervation patterns by uninjured CGRP fibers in the rat foot sole epidermis after nerve injury. Mol Pain 2012;8:61. 22935198 10.1186/1744-8069-8-61 PMC3492210

[15] Dyer LA Pi X Patterson C The role of BMPs in endothelial cell function and dysfunction. Trends Endocrinol Metab 2014;25:472–80. 24908616 10.1016/j.tem.2014.05.003 PMC4149816

[16] Eisenman ST Gibbons SJ Singh RD Bernard CE Wu J Sarr MG Kendrick ML Larson DW Dozois EJ Shen KR Farrugia G Distribution of TMEM100 in the mouse and human gastrointestinal tract—a novel marker of enteric nerves. Neuroscience 2013;240:117–28. 23485812 10.1016/j.neuroscience.2013.02.034 PMC3637859

[17] Engle MP Merrill MA Marquez De Prado B Hammond DL Spinal nerve ligation decreases gamma-aminobutyric acid B receptors on specific populations of immunohistochemically identified neurons in L5 dorsal root ganglion of the rat. J Comp Neurol 2012;520:1663–77. 22120979 10.1002/cne.23005 PMC3902039

[18] Ferreira-Gomes J Adaes S Sarkander J Castro-Lopes JM Phenotypic alterations of neurons that innervate osteoarthritic joints in rats. Arthritis Rheum 2010;62:3677–85. 20722015 10.1002/art.27713

[19] Fischer G Kostic S Nakai H Park F Sapunar D Yu H Hogan Q Direct injection into the dorsal root ganglion: technical, behavioral, and histological observations. J Neurosci Methods 2011;199:43–55. 21540055 10.1016/j.jneumeth.2011.04.021 PMC3742008

[20] Fischer G Pan B Vilceanu D Hogan QH Yu H Sustained relief of neuropathic pain by AAV-targeted expression of CBD3 peptide in rat dorsal root ganglion. Gene Ther 2014;21:44–51. 24152582 10.1038/gt.2013.56 PMC3881029

[21] Frullanti E Colombo F Falvella FS Galvan A Noci S De Cecco L Incarbone M Alloisio M Santambrogio L Nosotti M Tosi D Pastorino U Dragani TA Association of lung adenocarcinoma clinical stage with gene expression pattern in noninvolved lung tissue. Int J Cancer 2012;131:E643–648. 22223368 10.1002/ijc.27426

[22] Fuchs A Rigaud M Sarantopoulos CD Filip P Hogan QH Contribution of calcium channel subtypes to the intracellular calcium signal in sensory neurons: the effect of injury. Anesthesiology 2007;107:117–27. 17585223 10.1097/01.anes.0000267511.21864.93 PMC3720140

[23] Gardell LR Vanderah TW Gardell SE Wang R Ossipov MH Lai J Porreca F Enhanced evoked excitatory transmitter release in experimental neuropathy requires descending facilitation. J Neurosci 2003;23:8370–9. 12967999 10.1523/JNEUROSCI.23-23-08370.2003 PMC6740686

[24] Gregory NS Harris AL Robinson CR Dougherty PM Fuchs PN Sluka KA An overview of animal models of pain: disease models and outcome measures. J Pain 2013;14:1255–69. 24035349 10.1016/j.jpain.2013.06.008 PMC3818391

[25] Hammond DL Ackerman L Holdsworth R Elzey B Effects of spinal nerve ligation on immunohistochemically identified neurons in the L4 and L5 dorsal root ganglia of the rat. J Comp Neurol 2004;475:575–89. 15236238 10.1002/cne.20209

[26] Han Z Wang T Han S Chen Y Chen T Jia Q Li B Li B Wang J Chen G Liu G Gong H Wei H Zhou W Liu T Xiao J Low-expression of TMEM100 is associated with poor prognosis in non-small-cell lung cancer. Am J Transl Res 2017;9:2567–78. 28560005 PMC5446537

[27] Hasel P Dando O Jiwaji Z Baxter P Todd AC Heron S Márkus NM McQueen J Hampton DW Torvell M Tiwari SS McKay S Eraso-Pichot A Zorzano A Masgrau R Galea E Chandran S Wyllie DJA Simpson TI Hardingham GE Neurons and neuronal activity control gene expression in astrocytes to regulate their development and metabolism. Nat Commun 2017;8:15132. 28462931 10.1038/ncomms15132 PMC5418577

[28] He Z Han D Efimova O Guijarro P Yu Q Oleksiak A Jiang S Anokhin K Velichkovsky B Grünewald S Khaitovich P Comprehensive transcriptome analysis of neocortical layers in humans, chimpanzees and macaques. Nat Neurosci 2017;20:886–95. 28414332 10.1038/nn.4548

[29] Honore P Rogers SD Schwei MJ Salak-Johnson JL Luger NM Sabino MC Clohisy DR Mantyh PW Murine models of inflammatory, neuropathic and cancer pain each generates a unique set of neurochemical changes in the spinal cord and sensory neurons. Neuroscience 2000;98:585–98. 10869852 10.1016/s0306-4522(00)00110-x

[30] Huang D Liang C Zhang F Men H Du X Gamper N Zhang H Inflammatory mediator bradykinin increases population of sensory neurons expressing functional T-type Ca(2+) channels. Biochem Biophys Res Commun 2016;473:396–402. 26944020 10.1016/j.bbrc.2016.02.118 PMC4840015

[31] Kikuchi S Chen L Xiong K Saito Y Azuma N Tang G Sobel M Wight TN Kenagy RD Smooth muscle cells of human veins show an increased response to injury at valve sites. J Vasc Surg 2017;67:1556–70.e9. 28647196 10.1016/j.jvs.2017.03.447 PMC5740028

[32] LaCroix-Fralish ML Austin JS Zheng FY Levitin DJ Mogil JS Patterns of pain: meta-analysis of microarray studies of pain. PAIN 2011;152:1888–98. 21561713 10.1016/j.pain.2011.04.014

[33] Li Q Lau A Morris TJ Guo L Fordyce CB Stanley EF A syntaxin 1, Galpha(o), and N-type calcium channel complex at a presynaptic nerve terminal: analysis by quantitative immunocolocalization. J Neurosci 2004;24:4070–81. 15102922 10.1523/JNEUROSCI.0346-04.2004 PMC6729428

[34] Li Y Dorsi MJ Meyer RA Belzberg AJ Mechanical hyperalgesia after an L5 spinal nerve lesion in the rat is not dependent on input from injured nerve fibers. PAIN 2000;85:493–502. 10781924 10.1016/S0304-3959(00)00250-5

[35] Liu Z Wang F Fischer G Hogan QH Yu H Peripheral nerve injury induces loss of nociceptive neuron-specific Galphai-interacting protein in neuropathic pain rat. Mol Pain 2016;12. 10.1177/1744806916646380 PMC4956147 27145804

[36] Lu SG Gold MS Inflammation-induced increase in evoked calcium transients in subpopulations of rat dorsal root ganglion neurons. Neuroscience 2008;153:279–88. 18367340 10.1016/j.neuroscience.2008.02.006 PMC2396945

[37] Luchting B Heyn J Woehrle T Rachinger-Adam B Kreth S Hinske LC Azad SC Differential expression of P2X7 receptor and IL-1beta in nociceptive and neuropathic pain. J Neuroinflammation 2016;13:100. 27145808 10.1186/s12974-016-0565-z PMC4857287

[38] Marques S Zeisel A Codeluppi S van Bruggen D Mendanha Falcao A Xiao L Li H Haring M Hochgerner H Romanov RA Gyllborg D Munoz-Manchado AB La Manno G Lonnerberg P Floriddia EM Rezayee F Ernfors P Arenas E Hjerling-Leffler J Harkany T Richardson WD Linnarsson S Castelo-Branco G Oligodendrocyte heterogeneity in the mouse juvenile and adult central nervous system. Science 2016;352:1326–9. 27284195 10.1126/science.aaf6463 PMC5221728

[39] McCallum JB Kwok WM Sapunar D Fuchs A Hogan QH Painful peripheral nerve injury decreases calcium current in axotomized sensory neurons. Anesthesiology 2006;105:160–8. 16810008 10.1097/00000542-200607000-00026 PMC3725035

[40] Mogil JS Animal models of pain: progress and challenges. Nat Rev Neurosci 2009;10:283–94. 19259101 10.1038/nrn2606

[41] Moon EH Kim MJ Ko KS Kim YS Seo J Oh SP Lee YJ Generation of mice with a conditional and reporter allele for Tmem100. Genesis 2010;48:673–8. 20848592 10.1002/dvg.20674

[42] Moon EH Kim YS Seo J Lee S Lee YJ Oh SP Essential role for TMEM100 in vascular integrity but limited contributions to the pathogenesis of hereditary haemorrhagic telangiectasia. Cardiovasc Res 2015;105:353–60. 25538155 10.1093/cvr/cvu260 PMC6279201

[43] Nguyen MQ Wu Y Bonilla LS von Buchholtz LJ Ryba NJP Diversity amongst trigeminal neurons revealed by high throughput single cell sequencing. PLoS One 2017;12:e0185543. 28957441 10.1371/journal.pone.0185543 PMC5619795

[44] Obata K Katsura H Mizushima T Yamanaka H Kobayashi K Dai Y Fukuoka T Tokunaga A Tominaga M Noguchi K TRPA1 induced in sensory neurons contributes to cold hyperalgesia after inflammation and nerve injury. J Clin Invest 2005;115:2393–401. 16110328 10.1172/JCI25437 PMC1187934

[45] Ou D Yang H Hua D Xiao S Yang L Novel roles of TMEM100: inhibition metastasis and proliferation of hepatocellular carcinoma. Oncotarget 2015;6:17379–90. 25978032 10.18632/oncotarget.3954 PMC4627315

[46] Pan B Yu H Fischer GJ Kramer JM Hogan QH Dorsal root ganglionic field stimulation relieves spontaneous and induced neuropathic pain in rats. J Pain 2016;17:1349–58. 27687223 10.1016/j.jpain.2016.09.004

[47] Peacock HM Caolo V Jones EA Arteriovenous malformations in hereditary haemorrhagic telangiectasia: looking beyond ALK1-NOTCH interactions. Cardiovasc Res 2016;109:196–203. 26645978 10.1093/cvr/cvv264

[48] Price TJ Flores CM Critical evaluation of the colocalization between calcitonin gene-related peptide, substance P, transient receptor potential vanilloid subfamily type 1 immunoreactivities, and isolectin B4 binding in primary afferent neurons of the rat and mouse. J Pain 2007;8:263–72. 17113352 10.1016/j.jpain.2006.09.005 PMC1899162

[49] Ramer MS Duraisingam I Priestley JV McMahon SB Two-tiered inhibition of axon regeneration at the dorsal root entry zone. J Neurosci 2001;21:2651–60. 11306618 10.1523/JNEUROSCI.21-08-02651.2001 PMC6762521

[50] Rouwette T Sondermann J Avenali L Gomez-Varela D Schmidt M Standardized profiling of the membrane-enriched Proteome of mouse dorsal root ganglia (DRG) provides novel insights into chronic pain. Mol Cell Proteomics 2016;15:2152–68. 27103637 10.1074/mcp.M116.058966 PMC5083083

[51] Sapunar D Modric-Jednacak K Grkovic I Michalkiewicz M Hogan QH Effect of peripheral axotomy on pain-related behavior and dorsal root ganglion neurons excitability in NPY transgenic rats. Brain Res 2005;1063:48–58. 16259969 10.1016/j.brainres.2005.09.019

[52] Shin J Berg DA Zhu Y Shin JY Song J Bonaguidi MA Enikolopov G Nauen DW Christian KM Ming GL Song H Single-cell RNA-seq with waterfall reveals molecular cascades underlying adult neurogenesis. Cell Stem Cell 2015;17:360–72. 26299571 10.1016/j.stem.2015.07.013 PMC8638014

[53] Somekawa S Imagawa K Hayashi H Sakabe M Ioka T Sato GE Inada K Iwamoto T Mori T Uemura S Nakagawa O Saito Y Tmem100, an ALK1 receptor signaling-dependent gene essential for arterial endothelium differentiation and vascular morphogenesis. Proc Natl Acad Sci U S A 2012;109:12064–9. 22783020 10.1073/pnas.1207210109 PMC3409742

[54] Staton PC Wilson AW Bountra C Chessell IP Day NC Changes in dorsal root ganglion CGRP expression in a chronic inflammatory model of the rat knee joint: differential modulation by rofecoxib and paracetamol. Eur J Pain 2007;11:283–9. 16690336 10.1016/j.ejpain.2006.03.006

[55] Stein C Millan MJ Herz A Unilateral inflammation of the hindpaw in rats as a model of prolonged noxious stimulation: alterations in behavior and nociceptive thresholds. Pharmacol Biochem Behav 1988;31:445–51. 3244721 10.1016/0091-3057(88)90372-3

[56] Trevisan G Benemei S Materazzi S De Logu F De Siena G Fusi C Fortes Rossato M Coppi E Marone IM Ferreira J Geppetti P Nassini R TRPA1 mediates trigeminal neuropathic pain in mice downstream of monocytes/macrophages and oxidative stress. Brain 2016;139(pt 5):1361–77. 26984186 10.1093/brain/aww038

[57] Vallès A Boender AJ Gijsbers S Haast RA Martens GJ de Weerd P Genomewide analysis of rat barrel cortex reveals time- and layer-specific mRNA expression changes related to experience-dependent plasticity. J Neurosci 2011;31:6140–58. 21508239 10.1523/JNEUROSCI.6514-10.2011 PMC6632955

[58] Wang F Xiang H Fischer G Liu Z Dupont MJ Hogan QH Yu H HMG-CoA synthase isoenzymes 1 and 2 localize to satellite glial cells in dorsal root ganglia and are differentially regulated by peripheral nerve injury. Brain Res 2016;1652:62–70. 27671501 10.1016/j.brainres.2016.09.032 PMC5441544

[59] Weng HJ Patel KN Jeske NA Bierbower SM Zou W Tiwari V Zheng Q Tang Z Mo GC Wang Y Geng Y Zhang J Guan Y Akopian AN Dong X Tmem100 is a regulator of TRPA1-TRPV1 complex and contributes to persistent pain. Neuron 2015;85:833–46. 25640077 10.1016/j.neuron.2014.12.065 PMC4336228

[60] Weyer AD Stucky CL Loosening pain's grip by tightening TRPV1-TRPA1 interactions. Neuron 2015;85:661–3. 25695265 10.1016/j.neuron.2015.02.004

[61] Wu HE Gemes G Zoga V Kawano T Hogan QH Learned avoidance from noxious mechanical simulation but not threshold semmes weinstein filament stimulation after nerve injury in rats. J Pain 2010;11:280–6. 19945356 10.1016/j.jpain.2009.07.011 PMC2891524

[62] Xiang H Xu H Fan F Shin SM Hogan QH Yu H Glial fibrillary acidic protein promoter determines transgene expression in satellite glial cells following intraganglionic adeno-associated virus delivery in adult rats. J Neurosci Res 2018;96:436–48. 28941260 10.1002/jnr.24183 PMC5766685

[63] Xu P Van Slambrouck C Berti-Mattera L Hall AK Activin induces tactile allodynia and increases calcitonin gene-related peptide after peripheral inflammation. J Neurosci 2005;25:9227–35. 16207882 10.1523/JNEUROSCI.3051-05.2005 PMC6725762

[64] Xu Q Yaksh TL A brief comparison of the pathophysiology of inflammatory versus neuropathic pain. Curr Opin Anaesthesiol 2011;24:400–7. 21659872 10.1097/ACO.0b013e32834871df PMC3290396

[65] Yamazaki T Muramoto M Okitsu O Morikawa N Kita Y Discovery of a novel neuroprotective compound, AS1219164, by high-throughput chemical screening of a newly identified apoptotic gene marker. Eur J Pharmacol 2011;669:7–14. 21824470 10.1016/j.ejphar.2011.07.027

[66] Ye P Hua L Jiao Y Li Z Qin S Fu J Jiang F Liu T Ji Y Functional up-regulation of Nav1.8 sodium channel on dorsal root ganglia neurons contributes to the induction of scorpion sting pain. Acta Biochim Biophys Sin (Shanghai) 2016;48:132–44. 26764239 10.1093/abbs/gmv123

[67] You HJ Park HY Kim J Lee IH Seol HJ Lee JI Kim ST Kong DS Nam DH Integrative radiogenomic analysis for genomic signatures in glioblastomas presenting leptomeningeal dissemination. Medicine (Baltimore) 2016;95:e4109. 27399113 10.1097/MD.0000000000004109 PMC5058842

[68] Yu H Fischer G Ebert AD Wu HE Bai X Hogan QH Analgesia for neuropathic pain by dorsal root ganglion transplantation of genetically engineered mesenchymal stem cells: initial results. Mol Pain 2015;11:5. 25888914 10.1186/s12990-015-0002-9 PMC4331376

[69] Yu H Fischer G Ferhatovic L Fan F Light AR Weihrauch D Sapunar D Nakai H Park F Hogan QH Intraganglionic AAV6 results in efficient and long-term gene transfer to peripheral sensory nervous system in adult rats. PLoS One 2013;8:e61266. 23613824 10.1371/journal.pone.0061266 PMC3628918

[70] Yu H Fischer G Jia G Reiser J Park F Hogan QH Lentiviral gene transfer into the dorsal root ganglion of adult rats. Mol Pain 2011;7:63. 21861915 10.1186/1744-8069-7-63 PMC3179738

[71] Yu L Yang F Luo H Liu FY Han JS Xing GG Wan Y The role of TRPV1 in different subtypes of dorsal root ganglion neurons in rat chronic inflammatory nociception induced by complete Freund's adjuvant. Mol Pain 2008;4:61. 19055783 10.1186/1744-8069-4-61 PMC2628345

[72] Zollner C Shaqura MA Bopaiah CP Mousa S Stein C Schafer M Painful inflammation-induced increase in mu-opioid receptor binding and G-protein coupling in primary afferent neurons. Mol Pharmacol 2003;64:202–10. 12869624 10.1124/mol.64.2.202

